# Quality of Life and Capsaicin Sensitivity in Patients with Airway Symptoms Induced by Chemicals and Scents: A Longitudinal Study

**DOI:** 10.1289/ehp.9624

**Published:** 2006-12-19

**Authors:** Ewa Ternesten-Hasséus, Olle Lowhagen, Eva Millqvist

**Affiliations:** Asthma and Allergy Research Group, Department of Respiratory Medicine and Allergology, The Sahlgrenska Academy at Göteborg University, Göteborg, Sweden

**Keywords:** asthma, capsaicin, cough, environment, health-related quality of ife, multiple chemical sensitivity, sensory hyperreactivity

## Abstract

**Objective:**

It is common in asthma and allergy clinics to see patients presenting with upper and lower airway symptoms that are induced by chemicals and scents and not explained by allergic or asthmatic reactions. Previous studies have shown that these patients often have increased cough sensitivity to inhaled capsaicin; such sensitivity is known to reflect the airway sensory reactivity. The aim of this study was to evaluate the duration of symptoms induced by chemicals and scents and to measure health-related quality of life (HRQL) in patients with chemically induced airway symptoms. We also wished to determine and compare repeatability of the cough response to capsaicin inhalation, and to evaluate the patients’ airway sensory reactivity in a long-term perspective.

**Participants:**

Seventeen patients with a history of at least 12 months of airway symptoms induced by chemicals and scents were followed over 5 years with repeated questionnaires, measurements of HRQL, and capsaicin inhalation tests.

**Results:**

The symptoms persisted and did not change significantly over time, and the patients had a reduced HRQL that did not change during the 5-year period. The capsaicin sensitivity was increased at the start of the study, the cough sensitivity was long-lasting, and the repeatability of the capsaicin inhalation test was considered to be good in a long-term perspective.

**Conclusions:**

Upper and lower airway symptoms induced by chemicals and scents represent an entity of chronic diseases, different from asthma or chronic obstructive pulmonary disease, with persistent symptoms, a reduced HRQL, and unchanged sensory hyperreactivity.

Previous studies have described patients with upper and lower airway symptoms induced by chemicals and scents (Johansson et al. 2006; Millqvist 2000; Millqvist et al. 1998; Ternesten-Hasseus et al. 2002a, 2002b). Inducing factors can include perfume, flowers, cleaning agents, car-exhaust fumes, and cigarette smoke. Steroids, β_2_-agonists, and other pharmacologic treatment for asthma or allergy have weak effects or none at all. Common symptoms are heavy breathing, difficulty getting air, cough, hoarseness, phlegm, nasal blockage, rhinorrhea, and eye irritation. The symptoms are often misinterpreted as asthma and/or allergy. Sensory hyperreactivity (SHR) is one suggested explanation for these airway symptoms induced by chemicals and scents (Johansson et al. 2002; Millqvist et al. 1998). The diagnostic criteria for the proposed diagnosis of SHR include self-reported sensitivity to chemicals and scents and a positive reaction to a capsaicin inhalation test, with the cut-off values described by Johansson et al. (2002). The prevalence of SHR has been estimated at 6.3% (Johansson et al. 2006).

There is a strong resemblance between SHR and multiple chemical sensitivity (MCS), but the diagnosis of SHR implies that just one organ is affected—the airways—whereas in MCS, symptoms from multiple organs are involved. However, some patients with SHR also complain of more general symptoms, such as sweating, dizziness, headache, and fatigue, bordering on a diagnosis of MCS (Ternesten-Hasseus et al. 2002a). MCS is also known as “the 20th century disease,” “chemically acquired immunodeficiency syndrome,” “total allergy syndrome,” and “idiopathic environmental intolerances” (American Academy of Allergy, Asthma and Immunology Board of Directors 1999; Graveling et al. 1999). MCS is associated with a generalized sensitivity to low doses of chemicals (normally regarded as nontoxic) in the environment, with symptoms from various organs (including the airways) and often non-specific symptoms such as fatigue and cognitive difficulties. Cullen (1987) defined the condition according to its major features. The definition of MCS may cover a number of conditions in patients with various symptoms and undiagnosed disorders.

Capsaicin, which stimulates the unmyelinated C-fibers of the sensory nervous system, has long been used to assess cough sensitivity related to airway sensory nerves (Fuller et al. 1985; Karlsson et al. 1988; Midgren et al. 1992; Morice and Geppetti 2004). Inhalation of capsaicin induces cough in a safe and dose-dependent manner, and it has good short-and long-term repeatability (Dicpinigaitis 2003; Dicpinigaitis and Alva 2005; Millqvist et al. 1998; Ternesten-Hasseus et al. 2006). However, the long-term repeatability of capsaicin cough challenge has only been studied in healthy subjects (Dicpinigaitis 2003). Patients with airway symptoms induced by chemicals and scents react more strongly than healthy controls and asthmatic patients to inhaled capsaicin (Johansson et al. 2002; Millqvist et al. 1998; Ternesten-Hasseus et al. 2002b), supporting the hypothesis that the explanation seems to be an overreaction of the sensory nervous system of the airways followed by a hyperreactivity to chemicals and scents—a sensory hyperreactivity. Increased cough sensitivity to inhaled capsaicin has also been found in MCS patients with airway symptoms (Nogami et al. 2004; Ternesten-Hasseus et al. 2002a). Previously, significantly increased levels of nerve growth factor in nasal lavage after capsaicin inhalation have been found in patients with SHR, suggesting a neurochemical imbalance of the respiratory system in these patients (Millqvist et al. 2005).

Health-related quality of life (HRQL) is a measure of how diseases and symptoms affect health, well being, and the ability to function in daily life. The Nottingham Health Profile (NHP) questionnaire was developed at Nottingham University (UK) for measuring subjective health status and has been used in several studies of various chronic illnesses and conditions (Löth et al. 1998; Ringsberg et al. 1990; Tsukino et al. 1996; Wiklund et al. 1991). Bilingual health care personnel have translated the NHP from English to Swedish, with the aim of expressing how patients experience the effects of illness, as stated in their own words. MCS has been found to negatively affect HRQL (Black et al. 1999), but there are few studies describing HRQL in patients with SHR (Millqvist et al. 2000).

The aims of the present study were to *a*) evaluate the duration of symptoms and to measure HRQL in patients with airway symptoms induced by chemicals and scents; *b*) determine and compare repeatability of the cough response to capsaicin inhalation; and *c*) evaluate the patients’ sensory reactivity in a long-term perspective.

## Materials and Methods

### Patients

The study group included 17 patients (13 women and 4 men; median age of 48 years) who were consecutively selected over a period of about 9 months, when they were referred to our outpatient asthma and allergy clinic because of symptoms suggestive of asthma or allergies accompanied by treatment failure. They were screened using a questionnaire on airway symptoms and symptoms in response to chemicals and scents. The patients had at least a 1 year (median 9 years) history of upper and lower airway symptoms induced by chemicals and scents. Within the last 3 years, all patients had had a skin prick test (SPT) with a standard panel of 10 allergen sources common to Sweden. The SPT was negative for all of the patients except for two who had a minor positive reaction to mugwort, which was regarded as being without clinical importance. The methacholine test, administered according to the method described by Lowhagen (1984), was negative for all patients. None of the patients demonstrated spirometric reversibility or variability in pulmonary function. Ten of the patients had never smoked, six had ceased smoking > 2 years before the study began, and one had ceased smoking 2 months previously. All participants except one were tested in a standardized way with the 1-butanol threshold test and the Scandinavian Odor-Identification Test (SOIT) (Cain et al. 1988; Nordin et al. 1998). All had a normal sense of smell according to the SOIT, although three had hyposmia (a reduced ability to smell and to detect odors) according to the 1-butanol test. Demographic data of the patients are presented in Table 1.

Informed consent was obtained from all patients. The study was approved by the Regional Ethical Review Board of Göteborg, Sweden.

### Study design

The patients were followed over a period of approximately 5 years. The patients saw their ordinary physician during the follow-up time, and we did not interfere in their treatment. During the first year, patients visited the clinic four times for a capsaicin inhalation test. They then visited the clinic approximately once a year during the following 4 years. At each study visit, the patients underwent a capsaicin inhalation test (eight capsaicin tests in total). Before the first appointment, and once per year thereafter, the patients answered a local questionnaire and their HRQL was evaluated (six times in total). All of the questionnaires were handed out to the patients and answered before the capsaicin inhalation test. If a patient was unable to come to the clinic, the questionnaires were sent by mail, completed at home, and mailed back.

### Questionnaires

At the beginning of the study, the patients answered a local questionnaire about symptoms and triggering factors for symptoms. They were asked to score symptoms that they had experienced over the previous year on a scale of 0–3 (0 = no symptoms; 1 = mild symptoms; 2 = moderate symptoms; and 3 = severe symptoms). Fourteen symptoms were analyzed: heavy breathing, difficulty getting air, chest weight, cough, phlegm, throat irritation, hoarseness, nasal blockage, rhinorrhea, eye irritation, sweating, headache, dizziness, and fatigue. To allow test–retest evaluation of the symptom score questionnaire, the patients completed this questionnaire twice at the beginning of the study. The patients were also asked about their working capacity and the medication they used at the start of the study and then once each year.

### Health-related quality of life

HRQL was assessed using the NHP questionnaire, a generic, self-administered instrument for measuring various aspects of HRQL (Hunt and McEwen 1980). The questionnaire has been shown to have both high validity and high reliability (Hunt et al. 1981, 1984; Wiklund et al. 1988). It consists of two parts. Part I contains 38 items covering six aspects of HRQL, concerning the domains of physical mobility, pain, energy, sleep, social isolation, and emotional reactions. The response alternatives for each item are “yes” and “no,” depending on whether that item fits the individual’s current situation. The possible score for each dimension ranges from zero (no problems at all) to 100 (presence of all problems within the area). The weighting procedure is based on Thurstone’s “paired comparison” method (McKenna et al. 1981). Part II of the NHP contains seven questions, again with yes/no alternatives, concerning the impact of health problems on the individual’s social functioning in terms of paid employment, housework, social life, family life, sex life, hobbies, and holidays. For part II, the proportions of positive answers to each of the seven questions are calculated separately and compared with the reference values. The patients were asked to answer the questions according to their actual current condition.

### Reference values for NHP

We used norm and reference values distributed by age and sex for parts I and II of the NHP. These values are based on data from larger populations that are currently used in family practice (Hunt and Wiklund 1987).

### Capsaicin inhalation

We prepared a stock solution of capsaicin [Sigma-Aldrich Sweden AB, Stockholm, Sweden; 1 mmol/L in ethanol (99.5%)]. We then diluted the stock solution with 0.9% saline, containing 1% ethanol by volume, to prepare aqueous provocation solutions of 0.4, 2, and 10 μmol/L. The provocation solutions were prepared regularly and stored frozen. A capsaicin aqueous solution of 100 μmol/L retains total stability after 3 months of storage in a freezer (Ternesten-Hasseus et al. 2002b).

Capsaicin was nebulized (Pari Boy 36, type 37.0130; Paulritzau Pari-Werk GmbH, Starnberg, Germany) and inhaled through a mouthpiece (Pari Inhalierboy, no. 36.75) by tidal volume breathing; a nose clip was not used. Saline (1 mL) and the three increasing concentrations of capsaicin (0.4, 2, and 10 μmol/L) in a 1-mL solution were inhaled to completion, or for a maximum of 6 min, in order to induce coughing; this was followed by a 4-min rest period. The total number of evoked coughs was counted for 10 min from the onset of each inhaled capsaicin concentration. Cough registration was performed by the same counter using a tape recorder. The total duration of each capsaicin inhalation test was approximately 45 min. On the first three occasions, no limit was set on the number of coughs. In the following tests, however, out of consideration for the patients, provocation was stopped if one dose of capsaicin provoked >70 coughs. The forced expiratory volume in 1 sec (FEV_1_) was measured using a calibrated spirometer (Vitalograph, Buckingham, UK) before and after the inhalation test, and the highest of three values was recorded.

Capsaicin inhalation was not performed in patients who had experienced respiratory infections within 1 month before scheduled testing. All medication was withheld for at least 4 hr, and antihistamines were withheld for at least 5 days before the inhalation tests.

### Statistical analysis

We used the Wilcoxon signed-rank test for paired data and the Mann-Whitney *U*-test for nonpaired data. The test–retest reliability of the symptom score questionnaire was determined using Spearman’s rank correlation coefficient. For part I of the NHP, we used Friedman’s test to compare repeated measurements. Fisher’s exact test was used in part II for nonpaired data, and Cochran’s Q-test was used in part II for repeated measurements.

The repeatability of the capsaicin inhalation test was determined using the method of Bland and Altman (1986). In this method, repeatability is analyzed by taking repeated measurements on a series of subjects. The result is examined using a simple plot showing the results of one measurement against those of the other measurement for each subject, and a plot of the difference between the measurements against their mean. The coefficient of reproducibility is calculated as 2 standard deviations (SDs) from the mean of the difference in coughing.

A probability (*p*) value < 0.05 was considered statistically significant.

## Results

Each of the 17 subjects visited the clinic four times during the first year. Three patients declined to perform the capsaicin provocation in year 3, and four patients declined in years 4 and 5. The reasons for drop out were recurrent airway infections (subjects 1, 8, and 13), moving to another city (subject 7), fear of experiencing prolonged airway symptoms due to the provocation (subject 12), and pregnancy or expected pregnancy (subjects 11 and 17).

The median value of FEV_1_ was 96% of that predicted before the first capsaicin inhalation test, and no significant difference was found after the capsaicin provocations. During the 5-year follow-up, we found no significant changes in the median values of FEV_1_ compared with the initial baseline values.

### Questionnaires

Table 2 presents patient data about medications used at the first and the last visit of the study, reported symptoms at the first appointment in the study, and factors triggering these symptoms. During the 5-year follow-up, 3 patients were granted a disability pension, whereas 9 patients had problems with their working capacity because of their sensitivity to chemical agents; 6 of these patients were periodically on sick leave because of their symptoms, alone or together with other conditions (muscular problems in 3 patients). There were some individual variations in the symptom scores, but in the group as a whole, the patients reported persistent symptoms without statistically significant changes between the first and the last visit during the study (Table 3).

The test–retest reliability of the symptom score questionnaire was high (*r_s_* = 0.77; *p* < 0.01) indicating good reliability for the questionnaire.

### Health-related quality of life

**Figure 1:**
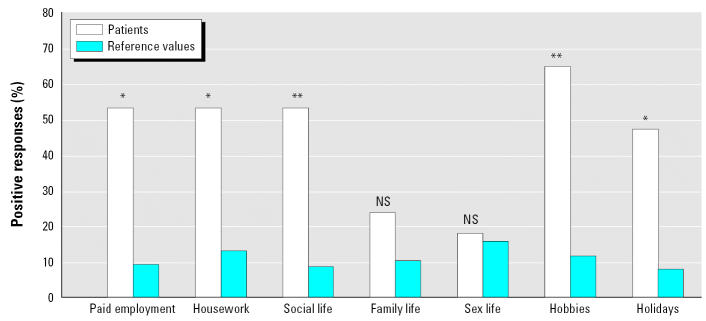
Percentage of positive responses reflecting problems in daily activity at the first appointment in year 1 in part II of the NHP for 17 patients and reference values. NS, not significant. *p < 0.05. **p < 0.01.

In the primary evaluation for part I of the NHP, patients had a significantly higher (*p* < 0.01) score for social isolation than the reference group. There were no statistically significant differences compared with the reference group in the other five dimensions (physical mobility, pain, energy, sleep, and emotional reactions). Part II of the NHP is presented in Figure 1 as a percentage of positive responses. The patients demonstrated significantly more problems than the reference group for five of the seven questions: paid employment (*p* < 0.05), housework (*p* < 0.05), social life (*p* < 0.01), hobbies (*p* < 0.01), and holidays (*p* < 0.05). Over the 4-year period the patients demonstrated no significant changes in any parts of the NHP compared with their initial evaluation.

### Capsaicin inhalation

**Figure 2:**
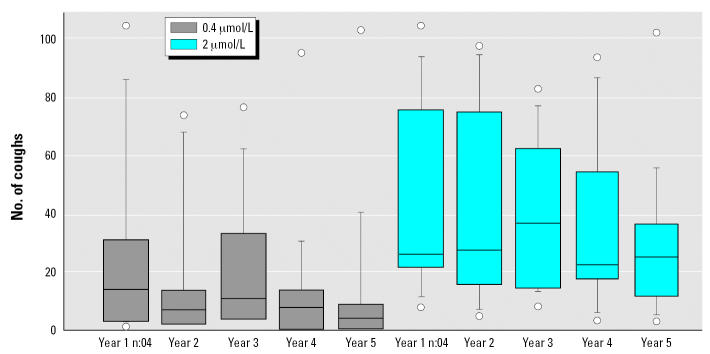
Box plot presentation of cough response after inhalation of two concentrations of capsaicin, 0.4 μmol/L and 2 μmol/L, on five separate occasions over 5 years. Year 1 n:04, fourth provocation in the first year. The horizontal line in the center of each box is the median; the top and bottom of the box represent the 25th and 75th percentiles; and whiskers indicate the 10th and 90th percentiles. Circles indicate individual maximum and minimum data points.

We counted the number of coughs by listening to the tape recordings. The patients coughed dose-dependently during the capsaicin provocations (Figure 2). Of the 17 patients, 15 had increased capsaicin cough sensitivity and reached the threshold set for the diagnosis of SHR according to Johansson et al. (2002). The inhalation was stopped for approximately 8 patients during each capsaicin inhalation test because they responded to one dose with > 70 coughs. Because there was no cough limit for the initial three provocations in the first year of the study, these results were not compared with the others. On each test occasion, many patients reached the cough limit at the highest dose of inhaled capsaicin (10 μmol/L); therefore, only results from the doses of 0.4 and 2 μmol/L are presented. We observed no significant differences between the numbers of coughs during the fourth provocation of the first year and the provocations of the second and third years. There was a significant decrease in the number of coughs between the fourth provocation of the first year and the provocations of the fourth and fifth years (*p* < 0.05).

**Figure 3:**
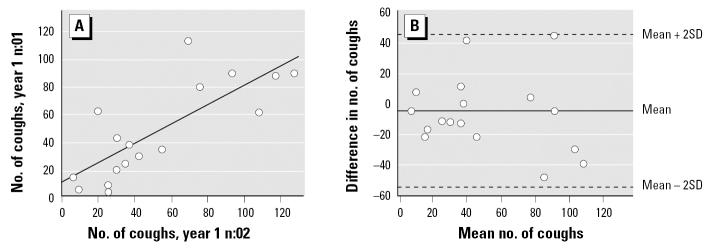
**(***A*) Repeatability of the number of coughs provoked by the inhalation of 2 μmol/L capsaicin in 17 patients on the first (n:01) and second (n:02) occasions of the first year (*r* = 0.78; *p* < 0.001). (*B*) Difference between the measurements on the first and second provocation occasions of the first year against their mean number of coughs on inhalation of 2 μmol/L capsaicin.

We calculated repeatability using a capsaicin concentration of 2 μmol/L. We compared the very first capsaicin provocation with the second provocation of the first year; the interval between these was about 3 months. The number of coughs after inhalation of 2 μmol/L capsaicin correlated significantly between these two provocation events (*r* = 0.78; *p* < 0.001) (Figure 3A). The SD from the mean difference between the 17 pairs of repeated measurements among the patients was 24 coughs for the 2-μmol/L capsaicin dose. The coefficient of repeatability was thus calculated to be 48 coughs (Figure 3B).

**Figure 4:**
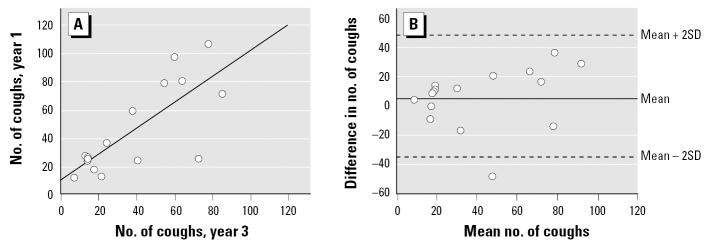
**(***A*) Repeatability of the number of coughs provoked by the inhalation of 2 μmol/L capsaicin in 15 patients on the last provocation of the first year and on the provocation of the third year (*r* = 0.76; *p* < 0.001). (*B*) Difference between the measurements on the last capsaicin provocation of the first year and on the provocation of the third year against their mean number of coughs on inhalation of 2 μmol/L capsaicin.

To evaluate the patients’ sensory reactivity in a longer perspective, we examined the repeatability of the capsaicin inhalation test using the same technique. We analyzed the two appointments in which the largest number (*n* = 15) of the same participants attended: the last provocation of the first year and the provocation of the third year. The number of coughs after inhalation of 2 μmol/L capsaicin correlated significantly between these two provocation occasions (*r* = 0.76; *p* < 0.001) (Figure 4A). The SD from the mean difference between the 15 pairs of repeated measurements among the patients was 21.5 coughs for the 2-μmol/L capsaicin dose. The coefficient of repeatability was thus calculated to be 43 coughs (Figure 4B).

## Discussion

In this 5-year study, we followed 17 patients with previously medically unexplained airway symptoms induced by chemicals and scents. These symptoms are often misdiagnosed as asthma or allergy, but they can be recognized using a capsaicin inhalation cough test (Johansson et al. 2002; Millqvist et al. 1998). According to this test, the majority of the patients in the present study (15 of 17) could be diagnosed with SHR. None of the patients recovered from their symptoms, and none developed asthma, chronic obstructive pulmonary disease, or any other known lung disease. After 5 years, brief exposure to substances such as perfume, colored paints, or cigarette smoke could still evoke symptoms, which had a long-term negative impact on the daily life of the patients. The patients reported long-lasting impairment in five of the seven activities in part II of the NHP (paid employment, housework, social life, hobbies, and holidays) compared with a population sample. They also reported significantly higher scores (more difficulties) for social isolation in part I of the NHP.

None of the patients in the present study demonstrated any serious adverse reaction or bronchoconstriction after the capsaicin inhalation test. In a recent review, Dicpinigaitis and Alva (2005) concluded capsaicin inhalation to be a safe research tool, with no serious adverse reactions reported over the past two decades. In two other studies with long-term occupational exposure to capsaicin, no demonstrable change in pulmonary function was seen (Blanc et al. 1991; Lankatilake and Uragoda 1993). Inhalation of three increments of capsaicin contains a total of 0.00377 g capsaicin, if the subject is able to inhale all three concentrations. This can be compared to a meal containing 100 g of fresh chili peppers, which would contain 0.1–1 g pure capsaicin. In some countries, consumption can be as high as approximately 3 g pure capsaicin per day (Govindarajan 1986). From this perspective, it seems unlikely that the capsaicin inhalation test could pose any risk to the patients.

Capsaicin-induced cough can, to a certain extent, be voluntarily suppressed or enhanced (Hutchings et al. 1993). Some individuals can avoid coughing when inhaling capsaicin, but the cough reflex becomes irresistible with increasing concentrations. In previous studies, inhalation of capsaicin induced a dose-dependent and reproducible cough in patients with SHR and healthy controls, when the capsaicin provocations were performed in a double-blind randomized fashion (Millqvist 2000; Ternesten-Hasseus et al. 2002a, 2006). With regard to the good long-term reproducibility, it seems unlikely that the patients could have intentionally influenced the outcome of the cough provocations. Although we found some individual changes in capsaicin cough sensitivity, the sensory reactivity among the patients was increased and long-standing.

Medically unexplained physical symptoms are common in clinical care and within the general population; examples include chronic fatigue syndrome, fibromyalgia, MCS, idiopathic environmental intolerances, somatoform disorders, and “Gulf War syndrome.” These symptoms are associated with extensive morbidity and have a significant impact on everyday functions (Baldwin et al. 1997; Black et al. 1999; Kipen et al. 1995; Kreutzer et al. 1999; Meggs et al. 1996). The association of these symptoms and syndromes with environmental exposure is often sharply debated, as is the distinction between the various syndromes themselves (Kipen and Fiedler 2002; Richardson and Engel 2004; Wiesmuller et al. 2003). However, in spite of the uncertainties in the evaluation of environmental syndromes, physicians have a duty to deal with the symptoms of those affected.

In conclusion, airway symptoms induced by chemicals and scents can be regarded as a chronic condition and may represent an entity of chronic diseases, with persistent symptoms, a reduced HRQL, and unchanged long-lasting sensory hyperreactivity. The repeatability for the capsaicin inhalation test is good in a short-term and long-term perspective. Although the capsaicin inhalation test was valuable for diagnosing these patients, the cause of their symptoms remains obscure, and there is currently no effective treatment; this emphasizes the need for future pathophysiologic research.

## Figures and Tables

**Table 1:** Demographic data for 17 patients with airway symptoms induced by chemicals and scents.

Patient no.	Sex	Age (years)	Duration of symptoms (years)	Baseline FEV_1_ (percent predicted)	Profession
1	F	45	3	95	Secretary/unemployed
2	M	42	2	87	Clerk
3	F	60	21	91	Disability pension
4	F	52	15	77	Nurse/periodically sick-listed
5	F	58	10	109	Teacher/periodically sick-listed
6	M	56	10	83	Clerk
7	M	48	8	88	Priest/periodically sick-listed
8	F	52	3	120	Nurse/periodically sick-listed
9	F	48	11	109	Occupational therapist’s assistant
10	F	37	4	95	Clerk
11	F	30	10	121	Statistician
12	F	31	15	131	Artist/unemployed
13	F	47	2	112	Postman/periodically sick-listed
14	M	61	5	91	Disability pension
15	F	53	1	106	Clerk
16	F	38	25	96	Salesman
17	F	27	2	78	Engineer/periodically sick-listed

Abbreviations: F, female; FEV_1_, forced expiratory volume in 1 sec; M, male.

**Table 2:** Number of patients reporting trigger factors, symptoms, and medication at the first and last visits in the study.

					No. of patients	
Trigger factors	No. of patients	Symptoms reported	No. of patients	Medication	First visit	Last visit
Flower scents	14	Heavy breathing	15	Inhalation steroids	1	1
Car exhaust fumes	13	Difficulty getting air	11	Nasal steroids	6	1
Perfume	16	Chest weight	14	Oral steroids	2	0
Cigarette smoke	16	Cough	17	Antihistamines	2	3
Colored paints	16	Phlegm	12	β_2_-Agonists	6	2
Exercise	15	Throat irritation	12	Antidepressant medication	2	2
Cold air	13	Hoarseness	13			
		Nasal blockage	10			
		Rhinorrhea	10			
		Eye irritation	15			
		Sweating	8			
		Headache	11			
		Dizziness	3			
		Fatigue	13			

**Table 3:** Severity (mean ± SD) of 14 symptoms in 17 patients in the first and last visits of the study, based on a score of 0–3.

Symptom	First visit	Last visit
Heavy breathing	1.35 ± 1.11	1.12 ± 0.78
Difficulty getting air	0.76 ± 0.90	0.59 ± 0.79
Chest weight	1.17 ± 1.18	0.82 ± 1.01
Cough	1.53 ± 1.12	1.70 ± 0.98
Phlegm	1.18 ± 1.07	1.29 ± 1.07
Throat irritation	1.09 ± 0.83	1.09 ± 0.98
Hoarseness	1.00 ± 1.27	0.70 ± 1.04
Nasal blockage	1.18 ± 1.33	1.23 ± 1.15
Rhinorrhea	1.24 ± 1.03	0.88 ± 0.92
Eye irritation	1.24 ± 1.09	0.88 ± 0.99
Sweating	0.88 ± 0.99	0.88 ± 0.99
Headache	1.18 ± 1.07	1.47 ± 1.23
Dizziness	0.29 ± 0.77	0.47 ± 0.72
Fatigue	1.64 ± 1.17	1.53 ± 1.28
